# A graphical tool for locating inconsistency in network meta-analyses

**DOI:** 10.1186/1471-2288-13-35

**Published:** 2013-03-09

**Authors:** Ulrike Krahn, Harald Binder, Jochem König

**Affiliations:** 1 Division Medical Biometry, Institute of Medical Biostatistics, Epidemiology and Informatics (IMBEI), University Medical Center Johannes Gutenberg University Mainz, Obere Zahlbacher Str. 69, 55131 Mainz, Germany

**Keywords:** Network meta-analysis, Inconsistency, Cochran’s Q, Hat matrix

## Abstract

**Background:**

In network meta-analyses, several treatments can be compared by connecting evidence from clinical trials that have investigated two or more treatments. The resulting trial network allows estimating the relative effects of all pairs of treatments taking indirect evidence into account. For a valid analysis of the network, consistent information from different pathways is assumed. Consistency can be checked by contrasting effect estimates from direct comparisons with the evidence of the remaining network. Unfortunately, one deviating direct comparison may have side effects on the network estimates of others, thus producing hot spots of inconsistency.

**Methods:**

We provide a tool, the net heat plot, to render transparent which direct comparisons drive each network estimate and to display hot spots of inconsistency: this permits singling out which of the suspicious direct comparisons are sufficient to explain the presence of inconsistency. We base our methods on fixed-effects models. For disclosure of potential drivers, the plot comprises the contribution of each direct estimate to network estimates resulting from regression diagnostics. In combination, we show heat colors corresponding to the change in agreement between direct and indirect estimate when relaxing the assumption of consistency for one direct comparison. A clustering procedure is applied to the heat matrix in order to find hot spots of inconsistency.

**Results:**

The method is shown to work with several examples, which are constructed by perturbing the effect of single study designs, and with two published network meta-analyses. Once the possible sources of inconsistencies are identified, our method also reveals which network estimates they affect.

**Conclusion:**

Our proposal is seen to be useful for identifying sources of inconsistencies in the network together with the interrelatedness of effect estimates. It opens the way for a further analysis based on subject matter considerations.

## Background

Evidence from various treatment comparisons in different randomized trials can be combined by a network meta-analysis. This method not only aggregates evidence from direct comparisons, but also involves indirect comparisons, i.e. relative effect inferences for previously observed or not observed contrasts. References [1-4] give an overview of the recent methodological development. The validity of a network meta-analysis and, in particular, that of the indirect comparisons depends on a consistent network of treatment effects. However, there might be specific treatment effects in the network that lead to inconsistency, e.g. due to being based on studies with divergent patient or trial characteristics [5,6] or due to bias [7]. Perturbed treatment effects can strongly affect other network estimates, which induces further inconsistency between direct and indirect estimates. This calls for tools that can identify the flow of evidence in the network, i.e. that can highlight direct comparisons that strongly drive other treatment effect estimates and hot spots of network inconsistency.

In this context, inconsistency means disagreement between direct and indirect evidence that can occur in addition to heterogeneity between studies with the same treatment arms. A network meta-analysis can be visualized by a graph, whereby the set of nodes corresponds to the considered treatments and the edges display the treatment comparisons of all included trials. If corresponding treatment effect estimates of various connections, or so called paths, differ between two treatments, there is inconsistency. Since the start and end point for different alternative network paths are the same, inconsistency can only be detected in such network loops [8,9]. It is not possible to trace inconsistency back to a single comparison in a network that only includes one loop, but comparisons that are included in several loops may be identifiable as a unique source for a hot spot of inconsistency.

In the following, we therefore provide methods for identifying such hot spots, which might consist of loops, parts of loops or even just single comparisons. We also investigate the influence of individual comparisons on the network estimates that might drive further perturbation and invalid network estimates due to the network design.

Different approaches to assess inconsistency have been discussed. The series of Technical Support Documents produced by the NICE Decision Support Unit [10] provides a detailed review of methods on this topic. The oldest method to assess inconsistency is to examine it in three-treatment loops [11]. For example, Cipriani et al. [12] apply it to every three-treatment loop in the network. While including larger loops as well, Salanti et al. [6] systematically repeat the method for every loop in the network. Another method to assess inconsistency is to set up a mixed model with a sparse covariance structure that allows for one extra variance component to capture inconsistency; this was performed in a classical likelihood framework [8] and in a Bayesian framework [9,13].

Finally, consistency can be assessed by comparing a model that satisfies only some consistency restrictions (or no restrictions at all) with the consistency model. The node-splitting method [14] extends the consistency model by only one parameter that captures the difference between a contrast, e.g. treatment *A* versus treatment *B*, that is assessed in all direct comparisons and the same contrast assumed to be valid from the indirect evidence. Unfortunately, the definition of the indirect evidence is not quite clear for multi-armed studies, and the node-splitting methods were recognized as depending on the choice of reference treatment in multi-armed studies [15,16]. Recently, Higgins et al. [15] and White et al. [16] have set up a modeling paradigm where studies are distinguished by design, i.e. by the full set of treatments compared. In this case, the effect of a contrast, e.g. between treatment *A* and treatment *B*, may differ in the full inconsistency model depending on being estimated in two-armed studies or e.g. in three-armed studies containing additionally treatment *C*, or treatment *D*. In their model, inconsistency is no longer a violation of some equations that reflect loops, but rather model parameters reflecting design-by-treatment interactions. Lu et al. [9] and White et al. [16] have used the term inconsistency degrees of freedom for the difference in the number of parameters between the full inconsistency model and the consistency model, but Lu et al. [9] defined them without distinguishing direct evidence from two-armed and multi-armed studies.

Lu and Ades [9] gave preference to a Bayesian approach and favored random-effects models that include inconsistency factors as random effects. Senn et al. [17] cautioned against random-effects analysis and pointed out (as did [18]) that in fixed-effects models with variances assumed to be known, a Cochran-type chi-squared statistic results for the overall heterogeneity in the network. Caldwell et al. [19] proposed a chi-squared statistic for testing the consistency of independent network paths between one pair of treatments. White et al. [16] proposed a global Wald chi-squared test for all design-by-treatment interaction parameters (treated as fixed effects), applied in a model with random effects for heterogeneity within designs that is fitted via restricted maximum likelihood method. This test may lack power by implicitly attributing part of the inconsistency to heterogeneity.

In this paper, we will define another global chi-squared test for inconsistency that results by comparing a fixed-effects model for inconsistency with a consistency model. It will emerge as a part of the decomposition of Cochran’s *Q* statistic into components accounting for heterogeneity among studies sharing the same design and inconsistency.

Once inconsistency has been assessed globally, means are needed to find its sources. Senn et al. [17] inspected the squared Pearson residuals at the study level, which sum up to the overall *Q* chi-squared statistic. The design-by-treatment interaction parameters introduced by [15,16] may be used to spot inconsistency. Unfortunately, the definition of these parameters relies on the ordering of treatments. More generally, all regression diagnostic methods (see [20]) can be applied. References [21,22] have discussed this for classical meta-analysis and [23] for network meta-analysis. There are some attempts to visualize network meta-analysis for assessing heterogeneity, including inconsistency [6,9,14,17,23,24]. None of these have met with general acceptance as yet, and they do not address the needs as well as the forest plot does in classical meta-analysis, which simultaneously discloses each study’s weight and deviation from the pooled estimate. Notably, for this purpose, the Galbraith plot [25], although far less commonly used, is even better suited.

In the following, we systematically develop a graphical tool for highlighting hot spots of inconsistency by considering the detailed change in inconsistency when detaching the effect of studies with the same treatment arms. Furthermore, we identify drivers for the network estimates. Highlighting of inconsistency will provide more information than just singling out inconsistent loops. We provide a matrix display that summarizes network drivers and inconsistency in two dimensions, such that it may be possible to trace inconsistency back to single deviating direct comparisons. Naturally, it is difficult to display detailed network properties in just two dimensions, but we propose a clustering approach that automatically groups comparisons for highlighting hot spots.

Section “Methods” provides a detailed description of the different building blocks of our proposal: We present a fixed-effects model for network meta-analyses within the framework of general linear models with known variances in Section “Parameterization and two-stage analysis of a fixed-effects model in network meta-analysis”. Based on this model, we discuss the resulting hat matrix in Section “Identifying drivers via the hat matrix”, which we use as an instrument for identifying drivers. We suggest using a chi-squared statistic for the heterogeneity in the network, which we decompose into a test statistic for the inconsistency and a test statistic for the heterogeneity within groups of studies, classified according to which treatments are involved. A graphical tool that visualizes the network drivers and inconsistency hot spots is given in Section “Identifying hot spots of inconsistency”. Specifically, we use the inconsistency information along with detaching of single component meta-analyses to locate inconsistency hot spots. All the steps in Section “Methods” are illustrated using artificial examples. Section “Results” then provides results for two published network analyses. Finally, we discuss our methods and results in Section “Discussion”, and we provide concluding remarks in Section “Conclusions”.

## Methods

In the following, we provide a fixed-effects model for network meta-analyses, on which we base our further analysis. We present tools to identify hot spots of inconsistency in the network and drivers with a high impact on network estimates. Using these two tools, we provide a graphical display to locate potential sources of inconsistency.

### Parameterization and two-stage analysis of a fixed-effects model in network meta-analysis

**Figure 1:**
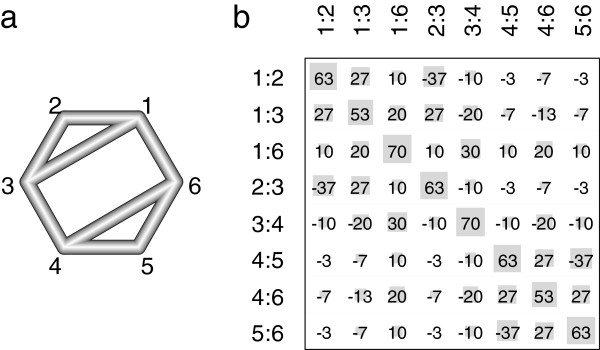
**Network design and hat matrix of an illustrative network meta-analysis.** In **a**), the network design of an illustrative example is given: six treatments and eight different observed designs based on two-armed studies. The nodes correspond to the treatments, and the edges show which treatments are directly compared. The thickness of an edge represents the inverse standard error ${\left(V_{d}^{\text{dir}}\right)}^{-1/2}$, which is equal one for all designs. In **b**), the resulting hat matrix at the design level is given in percent, which indicates the contribution of the direct estimate in design *d* (shown in the column) to the network estimate in design *d*’ (shown in the row). In addition, the absolute values of the matrix elements are visualized by the area of the gray squares.

We consider a network meta-analysis with *T*+1 treatments *A*_0_,…,*A*_*T*_, under which *A*_0_ represents a reference treatment. A total of *S* studies compares these treatments, such that a graphical representation of the comparison network with treatments as nodes and edges linking treatments directly compared in some studies creates a connected graph (see e.g. Figure 1a). We summarize all studies *s* (*s*=1,…,*S*) in the set S, classify each study by the number of included treatments *N*_*s*_ and by a design index *d*=1,…,*D* according to which treatments are respectively involved (see [15,16,23] for a similar approach). We define $S_{d}$ as the subset of studies with the same design *d* that includes *N*_*d*_ different treatments.

For a fixed-effects analysis, this network can be written in matrix notation as the following general linear model with heteroscedastic sampling variances:

(1) $$Y=X\theta^{\text{net}}+\mathrm{\in ;}$$

*Y* is a vector of observed treatment effects of all *S* studies, e.g. log odds ratios for a binary outcome and the design matrix *X* with *T* columns contains the structure of the network at the study level. For all studies of one design, we choose the same reference treatment. Assuming a consistent network, we estimate the vector of basic parameters *θ*^net^ (in terminology of [9]) corresponding to the treatment effects of all *T* comparisons to the reference treatment. By considering linear combinations of them, we can then infer all other effects of the network. The vector *∈* comprises all error terms of the model with *E*(*∈*)=0 and known covariance matrix *V*, which has a diagonal form. The length of vector *Y* and *∈* as well as the number of rows of *X* and *V* depend on the number and design of the included studies. Each two-armed study provides one entry to *Y*, one entry to the diagonal of *V*, and one row to *X*. We deal with the case of multi-armed studies separately in Section “Multi-armed studies”.

For exemplifying the model components, we consider a simple example of a network meta-analysis with three treatments *A*_0_,*A*_1_,*A*_2_ (*T*=2) and four observed studies (*S*=4): two studies (*s*=1,2) for comparison *A*_1_ versus *A*_0_ (*d*=1), one study (*s*=3) for comparison *A*_2_ versus *A*_0_ (*d*=2), and one study (*S*=4) for comparison *A*_2_ versus *A*_1_ (*d*=3). Then the basic parameters are $\theta_{0:1}^{\text{net}}$ and $\theta_{0:2}^{\text{net}}$ for the contrast of treatment *A*_1_ versus *A*_0_ (*d*=1, named 0:1) and the contrast of treatment *A*_2_ versus *A*_0_ (*d*=2, named 0:2). Under the consistency assumption, it follows that the effect $\theta_{1:2}^{\text{net}}=\theta_{0:2}^{\text{net}}-\theta_{0:1}^{\text{net}}$. Let *Y*_*s*_ be the observed effect and *V*_*s*_ is the corresponding sampling variance in study *s*. We then have:

$$Y\;=\;\left(\begin{array}{c} Y_{1}\\ Y_{2}\\ Y_{3}\\ Y_{4} \end{array}\right),\;X\;=\;\left[\begin{array}{cc} 1 & 0\\ 1 & 0\\ 0 & 1\\ -1 & 1 \end{array}\right],\;V\;=\;\left[\begin{array}{cccc} V_{1} & 0 & 0 & 0\\ 0 & V_{2} & 0 & 0\\ 0 & 0 & V_{3} & 0\\ 0 & 0 & 0 & V_{4} \end{array}\right].$$

The vector of the basic parameters *θ*^net^ can be estimated in a classical frequentist manner by generalized least squares as follows:

(2) $${\hat{\theta }}^{\text{net}}={\left(X^{\prime }V^{-1}X\right)}^{-1}X^{\prime }V^{-1}Y,$$

which is sometimes referred to as the Aitken estimator [26].

This estimation can equivalently be performed in two steps (as discussed in [18,23]). First, *D* meta-analyses with inverse variance weighting summarization provide pooled estimates and their variances per study design. Secondly, model (1) deals with the results of these component meta-analyses just as with single study observations. The inverse variance weighting estimation of the first step is as follows:

(3) $$\begin{array}{l} {\hat{\theta }}_{d}^{\text{dir}}:={\left(\underset{s\in ;S_{d}}{\sum }V_{s}^{-1}\right)}^{-1}\underset{s\in ;S_{d}}{\sum }V_{s}^{-1}Y_{s}\; \end{array}$$

(4) $$\begin{array}{l} V_{d}^{\text{dir}}:=\text{cov}\left({\hat{\theta }}_{d}^{\text{dir}}\right)={\left(\underset{s\in ;S_{d}}{\sum }V_{s}^{-1}\right)}^{-1}.\; \end{array}$$

Thus, evidence of all studies with the same treatment arms $\left(s\in ;S_{d}\right)$ is initially summarized, resulting in estimated treatment effects ${\hat{\theta }}_{d}^{\text{dir}}$ and covariance matrices $V_{d}^{\text{dir}}$ of so-called direct comparisons, since these comparisons are actually observed. In the second stage of the estimation, a linear model is fitted to the effect vector ${\hat{\theta }}^{\text{dir}}:=({\hat{\theta }}_{1}^{\text{dir}},\ldots ,{\hat{\theta }}_{D}^{\text{dir}})$’ of all summarized direct comparisons:

(5) $${\hat{\theta }}^{\text{dir}}=X_{a}\theta^{\text{net}}+\in_{a}$$

with *E*(*∈*_*a*_)=0 and *Cov*(*∈*_*a*_)=:*V*_*a*_. The covariance matrix is given by $V_{a}:=\text{diag}(V_{1}^{\text{dir}},\ldots ,V_{D}^{\text{dir}})$ and *X*_*a*_ is the compressed design matrix containing one set of rows for each design. In the case of two-armed studies, the design matrix *X*_*a*_ is formed by stacking one row over each of the other’s rows for each type of design. In the example above we have:

$$X_{a}=\left[\begin{array}{cc} 1 & 0\\ 0 & 1\\ -1 & 1 \end{array}\right].$$

#### Multi-armed studies

We distinguish each set of multi-armed studies sharing the same set of treatments as a different design. That means that if we add a three-armed study for *A*_2_ versus *A*_1_ versus *A*_0_ to the example above, we consider a further design (*d*=4).

Since the effects observed in one multi-armed study cannot be inconsistent, we use one design-specific treatment as a study reference for each multi-armed study, e.g. *A*_0_ in all studies comparing *A*_2_ versus *A*_1_ versus *A*_0_. Then, a study with *M*+1 arms adds to the vector *Y* of model (1) a vector *Y*_*s*_ of *M* treatment effects for each comparison to the reference. In our example we have the vector *Y*_*s*_=(*Y*_0:1_,*Y*_0:2_)’ of comparison *A*_1_ versus *A*_0_ and comparison *A*_2_ versus *A*_0_. Furthermore, the multi-armed study gives *M* rows for *X* with the corresponding contrasts. Since pairwise treatment effects of one study are correlated, the multi-armed study adds a block *V*_*s*_ of size *M*×*M* for the covariance matrix *V* of the sampling error *∈* (compare to [13,27]). In the case of multi-armed studies of design *d*, a summarized treatment effect ${\hat{\theta }}_{d}^{\text{dir}}$ is a vector of length *M* with covariance matrix $V_{d}^{\text{dir}}$ of size *M*×*M*. These summarizations can be calculated in accordance with the equations (3,4) [28] and can be used as observations in model (5). The design matrix *X*_*a*_ contains then *M* rows for the corresponding design of the studies. This means in the simple example above with four two-armed and one three-armed study (D=4) that:

$$X_{a}=\left[\begin{array}{cc} 1 & 0\\ 0 & 1\\ -1 & 1\\ 1 & 0\\ 0 & 1 \end{array}\right].$$

### Identifying drivers via the hat matrix

In linear models, the hat matrix contains the linear coefficients that present each predicted outcome as a function of all observations. Its diagonal elements are known as leverages. They summarize the importance of the respective observation for the whole estimation. Observations with both high leverage and large residual are recognized as being highly influential [29].

In the context of network meta-analyses and model (5), the hat matrix is:

(6) $$H:=X_{a}{\left(X_{a}^{\prime }V_{a}^{-1}X_{a}\right)}^{-1}X_{a}^{\prime }V_{a}^{-1}.$$

Its rows are the linear coefficients of ${\hat{\theta }}_{d}^{\text{dir}}$ (*d*=1,…,*D*) for the network estimate ${\hat{\theta }}_{d^{\prime }}^{\text{net}}$, where ${\hat{\theta }}_{d^{\prime }}^{\text{net}}$ is the subvector of ${\hat{\theta }}^{\text{net}}$ corresponding with design *d*’. The coefficients are a generalization of the study weights in simple meta-analyses but do not sum up to one. They have values between -1 and 1. While in simple meta-analyses the contribution of a study (or weight of a study) to the pooled estimate is proportional to the precision of the study, in network meta-analyses the contribution of direct estimates to a network estimate is a function not only of its precisions, but also of the network structure. Depending on the agreement of direct and indirect evidence, a large absolute entry in *H* indicates a strong influence of the respective direct estimate. Note that *H* is not necessarily symmetric, and for multi-armed studies the choice of the reference treatment affects the corresponding coefficients.

In network meta-analyses, the diagonal elements of *H* have a special role. In a connected network, the trace of *H* equals *T*, the number of parameters of model (5). In fact, each network estimate can be written as a weighted mean of a direct estimate which is based on all comparisons involving only the given two treatments and the indirect estimate which is based on all other studies. The diagonal element of *H* is identical to the weight of the direct estimate in this presentation. Different than in many regression applications, the off-diagonal elements of *H* deserve special attention in network meta-analyses. The smaller the diagonal element, the more weight is given to indirect evidence. This is also discussed in [18]. The off-diagonals indicate which study designs contribute in an essential way to the indirect part of the network estimate.

As an illustration of the hat matrix, we use an example of a network meta-analysis with six treatments (*T*=5) and eight different observed designs (*D*=8) based on two-armed studies (*N*_*d*_=2 for all *d*=1,…,8). The corresponding network is shown in Figure 1a), where the nodes correspond to the treatments and the edges show which treatments are directly compared. The thickness of an edge represents the inverse standard error (*Vd*dir)^−1/2^, which is equal to one for all *d* in our example (*V*_*a*_=*I*_8_, where *I*_8_ is the identity matrix of size eight). For one design there might, for example, be one study with $V_{s}^{-1/2}=1$ or 100 studies with $V_{s}^{-1/2}=0.1$. The resulting hat matrix at the design level is given in percent in Figure 1b). In addition, the absolute values of the matrix elements are visualized by the area of the gray squares.

The diagonal squares indicate that the network estimates are predominantly driven by their corresponding direct estimates, all more than 50%. The diagonal squares are the largest for the edges 1:6 and 3:4 that intercede between the two triangles. Their direct estimates drive 70% of their network estimates. The smallest diagonal squares are seen for the edges 1:3 and 4:6 (direct estimates drive 53%), since the latter ones are paralleled by two independent indirect paths and the former ones only by one. Inspecting the off-diagonal squares, we learn that aside from its direct estimates, the network estimates $\theta_{1:2}^{\text{net}}$ and $\theta_{2:3}^{\text{net}}$ are driven by the other corresponding direct estimate and then by $\theta_{1:3}^{\text{dir}}$. Due to symmetry, the same holds for the edges involved in the triangle {4:5, 5:6, 4:6}.

### Identifying hot spots of inconsistency

#### Decomposition of Cochran’s Q

An important aspect in meta-analysis is to investigate statistical heterogeneity. In network meta-analysis inconsistency arises as another aspect of heterogeneity. In a classical meta-analysis comparing two treatments, Cochran’s Q [30] is a well-accepted tool for assessing heterogeneity between studies, which is seen to be the sum of squared Pearson residuals. We use the generalized Cochran’s Q statistic for multivariate meta-analysis [27,31] in the context of network meta-analyses:

(7) $$Q^{\text{net}}:={\left(Y-X{\hat{\theta }}^{\text{net}}\right)}^{\prime }V^{-1}(Y-X{\hat{\theta }}^{\text{net}}).$$

To examine the heterogeneity of the whole network in more detail, particularly considering the inconsistency in the model, we decompose the *Q*^net^ statistic into two parts (similar to [32] who used a decomposition by study group in the context of classical meta-analysis):

(8) $$Q^{\text{net}}=Q^{\text{het}}+Q^{\text{inc}}.$$

The first is a sum of within-design Q statistics

(9) $$\begin{array}{ll} Q^{\text{het}} & :=\overset{D}{\underset{d=1}{\sum }}Q_{d}^{\text{het}}\text{with}\; \end{array}$$

(10) $$\begin{array}{ll} Q_{d}^{\text{het}} & :=\underset{s\in ;S_{d}}{\sum }{\left(Y_{s}-{\hat{\theta }}_{d}^{\text{dir}}\right)}^{\prime }V_{s}^{-1}(Y_{s}-{\hat{\theta }}_{d}^{\text{dir}}).\; \end{array}$$

The second is a between-designs Q statistic

(11) $$Q^{\text{inc}}:={\left({\hat{\theta }}^{\text{dir}}-X_{a}{\hat{\theta }}^{\text{net}}\right)}^{\prime }V_{a}^{-1}({\hat{\theta }}^{\text{dir}}-X_{a}{\hat{\theta }}^{\text{net}}).$$

The heterogeneity of the whole network can be assigned to the heterogeneity between studies by *Q*^het^, related to each design *d* with $Q_{d}^{\text{het}}$, and otherwise to the inconsistency of the network by *Q*^inc^. Under the null hypothesis for both homogeneity and consistency, all Q statistics ((7), (9), (10), (11)) are approximately chi-squared distributed with respective degrees of freedom given in Table 1. Thereby, the degrees of freedom of the chi-squared distribution corresponding to *Q*^inc^ are identical to those defined in [16]. All Q statistics are independent of the choice of design-specific reference treatment.

**Table 1:** The network Q statistics and the degrees of freedom of their corresponding chi-squared distribution

**Null hypothesis**	**Q statistic**	**Degrees of freedom**
Homogeneity in the whole network	*Q*^net^	$df_{Q^{\text{net}}}:=\left(\underset{s\in ;S}{\sum }(N_{s}-1)\right)-T$
Homogeneity within designs	*Q*^het^	$df_{Q^{\text{het}}}:=\overset{D}{\underset{d=1}{\sum }}df_{Q_{d}^{\text{het}}}$
Homogeneity within design *d*	$Q_{d}^{\text{het}}$	$df_{Q_{d}^{\text{het}}}:=\left(\underset{s\in ;S_{d}}{\sum }(N_{s}-1)\right)-N_{d}+1$
Consistency between designs	*Q*^inc^	$df_{Q^{\text{inc}}}:=\left(\overset{D}{\underset{d=1}{\sum }}(N_{d}-1)\right)-T$
Consistency between designs after	$Q_{\left(d\right)}^{\text{inc}}$	$df_{Q_{\left(d\right)}^{\text{inc}}}:=df_{Q^{\text{inc}}}-N_{d}+1$
detaching the effect of design *d*		

In a network with *T*+1 treatments and a set of studies S, *d*=1,…,*D* designs are observed. The set of studies with design *d* is given by $S_{d}$. The numbers of treatments in study *s* and in design *d* are given by *N*_*s*_ and *N*_*d*_ respectively.

For example, for the network design in Figure 1a) we assume inconsistent treatment effects by ${\left({\hat{\theta }}_{1:2}^{\text{dir}},{\hat{\theta }}_{1:3}^{\text{dir}},{\hat{\theta }}_{1:6}^{\text{dir}},\ldots ,{\hat{\theta }}_{5:6}^{\text{dir}}\right)}^{\prime }=(5,0,0,\ldots ,0)$’, where each component meta-analysis corresponds to one study. The perturbation effect of five means that the contrast differs by five standard errors of a direct effect estimate. This may be a lot if the precision of component meta-analysis is small. This effect was chosen here in order to achieve a reasonable power for illustration purposes.

In real applications, the power may be small [33] and a failure to detect inconsistency does not automatically imply consistency. Note, however, that a deviating effect cannot be absorbed into a heterogeneity variance component, other than in random-effects models. Depending on the number of studies that inform a design, a single deviating study may inflate either *Q*^inc^ or *Q*^het^. That is why inconsistency and heterogeneity must be considered jointly.

As network estimates, we obtain in the example ${\left({\hat{\theta }}_{1:2}^{\text{net}},{\hat{\theta }}_{1:3}^{\text{net}},{\hat{\theta }}_{1:6}^{\text{net}},{\hat{\theta }}_{2:3}^{\text{net}},{\hat{\theta }}_{3:4}^{\text{net}},{\hat{\theta }}_{4:5}^{\text{net}},{\hat{\theta }}_{4:6}^{\text{net}},{\hat{\theta }}_{5:6}^{\text{net}}\right)}^{\prime }=(3.167,1.333,0.500,-1.834,-0.500,-0.166,-0.333,-0.167)$’. With this, an inconsistency statistic *Q*^inc^=3.36+1.78+0.25+3.36+0.25+0.03+0.11+0.03=9.17 results that is chi-squared distributed with 8−5=3 degrees of freedom. Since there cannot be heterogeneity between studies, in this example *Q*^inc^ and $df_{Q^{\text{inc}}}$ are identical to *Q*^net^ and $df_{Q^{\text{net}}}$.

If some of the component meta-analyses are heterogeneous, the others can still validly be tested by their $Q_{d}^{\text{het}}.$ Even *Q*^inc^ has some interpretation in this case: The direct estimates are estimates of the inverse variance-weighted averages of different true but unknown study-specific treatment effects. Then, *Q*^inc^ with the same reference distribution provides a valid test of the hypothesis of consistency of these averaged treatment effects.

#### Detaching a single design

Once inconsistency is indicated by a large *Q*^inc^, formula (11) can be used to assess the contribution of each component meta-analysis of design *d* to the inconsistency. In fact, *Q*^inc^ is the sum of quadratic forms of residuals over all designs. For simple comparisons between two treatments, the summands are squared Pearson residuals. Unfortunately, a deviating effect of one component meta-analysis can simultaneously inflate several residuals. Therefore, we fit a set of extended models allowing for a deviating effect of each study design in turn and recalculate the *Q* statistic. This procedure is equivalent to a ‘leave one out’ approach: Once per fit, studies with one design are left out of the network estimate to obtain an independent estimate of the treatment effect in design *d* and to obtain a network model fit independent of studies with design *d* (for another leave one out approach, see [14]).

More formally, we modify model (5) by inserting *N*_*d*_−1 new parameters $\theta_{d}^{\text{dir-ind}}$ into the parameter vector $\theta_{\left(d\right)}^{\text{net}}:=(\theta^{\text{net}},\theta_{d}^{\text{dir-ind}})$ for all pairwise treatment comparisons in design *d* to the design-specific reference. The design matrix of the new model needs an extra column for each new parameter, here notated as set of indicator vectors $1_{\left\{d\right\}}$, with entry one for each pair-wise comparison in *d* and entry zero for all other comparisons. So we add *N*_*d*_−1 columns for each design with *N*_*d*_ treatments. Each additional column corresponds to one of the non-reference-treatments. We have the following model:

(12) $${\hat{\theta }}^{\text{dir}}=(X_{a},1_{\left\{d\right\}})\theta_{\left(d\right)}^{\text{net}}+\in ,$$

with *E*(*∈*)=0 and *Cov*(*∈*)=*V*_*a*_ as previously. In this model, the parameters *θ*^net^ capture all network evidence without the information from studies with design *d*, and the parameters $\theta_{d}^{\text{dir-ind}}$ denote the difference between direct and indirect effect estimate in design *d*. The latter is called a design-by-treatment interaction in White et al. [16], but in contrast to White et al., we only add extra columns for one design at a time. Remaining inconsistency in this model can be tested by the corresponding Q statistic:

(13) $$Q_{\left(d\right)}^{\text{inc}}:=R_{\left(d\right)}^{\prime }V_{a}^{-1}R_{\left(d\right)}$$

that is chi-squared distributed with $df_{Q_{\left(d\right)}^{\text{inc}}}$ degrees of freedom (see Table 1). Here, the vector

(14) $$R_{\left(d\right)}:={\hat{\theta }}^{\text{dir}}-(X_{a},1_{\left\{d\right\}}){\hat{\theta }}_{\left(d\right)}^{\text{net}}$$

of length $\overset{D}{\underset{d=1}{\sum }}(N_{d}-1)$ contains the residuals that are identical to those of a consistency model fitted after holding out design *d*. And in the case of design *d*, the residuals equal zero.

For illustration purposes, we successively introduce one new parameter for each of the eight possible detachments of one component meta-analysis into the inconsistent network example from Section “Decomposition of Cochran’s Q” corresponding to Figure 1. For design 1:2, we use a parameter vector extended by *θ*1:2dir-ind in model (12); in combination with the design matrix $\left(X_{a},{\left(1,0,\ldots ,0\right)}^{\prime }\right)$. With ${\hat{\theta }}_{\left(1:2\right)}^{\text{net}}=(5,0,\ldots ,0)$’ this results in $Q_{\left(1:2\right)}^{\text{inc}}=0+\cdots +0=0$ that is chi-squared distributed with two degrees of freedom. For design 1:3, we respectively obtain $Q_{\left(1:3\right)}^{\text{inc}}=1.15+0.00+1.15+1.15+1.15+0.13+0.50+0.13=5.36$ that is also chi-squared distributed with two degrees of freedom.

Finally, to locate the inconsistency in the network, we compare the remaining inconsistency after exclusion of design *d* studies to the inconsistency before exclusion for all designs *d*^′^=1,⋯,*D* by:

(15) $$Q_{d^{\prime },d}^{\text{diff}}:=Q_{d^{\prime \prime }}^{\text{inc}}-Q_{d^{\prime }(d)}^{\text{inc}}.$$

Here,

(16) $$Q_{d^{\prime }}^{\text{inc}}:={\left({\hat{\theta }}_{d^{\prime }}^{\text{dir}}-X_{d^{\prime }}{\hat{\theta }}^{\text{net}}\right)}^{\prime }V_{d^{\prime }}^{-1}({\hat{\theta }}_{d^{\prime }}^{\text{dir}}-X_{d^{\prime }}{\hat{\theta }}^{\text{net}})$$

is the summand in *Q*^inc^ belonging to design *d*’ (it is $\overset{D}{\underset{d^{\prime }=1}{\sum }}Q_{d^{\prime }}^{\text{inc}}=Q^{\text{inc}}$), and $Q_{d^{\prime }(d)}^{\text{inc}}$ is the corresponding part from $Q_{\left(d\right)}^{\text{inc}}$ and model (12). Since $R_{(d)d^{\prime }}=0$ for all *d*^′^=*d*; it follows that in this case $Q_{d^{\prime },d}^{\text{diff}}=Q_{d^{\prime }}^{\text{inc}}$. In other words, $Q_{d^{\prime },d}^{\text{diff}}$ is the reduction of the squared standardized residual $Q_{d^{\prime }}^{\text{inc}}$ for design *d*’ due to elimination of design *d* studies.

In the example, holding out design 1:2 results in a perfect fit of model (12) and we obtain $Q_{d^{\prime },1:2}^{\text{diff}}=Q_{d^{\prime }}^{\text{inc}}$ for all *d*’ in {1:2,...5:6} ( $Q_{1:2,1:2}^{\text{diff}}=3.36,Q_{1.3,1:2}^{\text{diff}}=1.78,\ldots \;$) since $Q_{d^{\prime }(1:2)}^{\text{inc}}=0$. For the detachment of the component meta-analysis with design 1:3, we obtain $Q_{1:2,1:3}^{\text{diff}}=2.21$, $Q_{1:3,1:3}^{\text{diff}}=1.78$, and so on.

#### The net heat plot

For a graphical inspection of network inconsistency, we use a color visualization of the quadratic matrix ${\left(Q_{d^{\prime },d}^{\text{diff}}\right)}_{d^{\prime },d=1,\ldots ,D}$, which we call a net heat plot in the following. Warm colors in this plot (yellow over orange to red) indicate a positive $Q_{d^{\prime },d}^{\text{diff}}$. A negative $Q_{d^{\prime },d}^{\text{diff}}$ is illustrated by blue colors. Because of the non-negative scalars $Q_{d^{\prime }}^{\text{inc}}$ on the diagonal of the matrix, which sum up to the *Q*^inc^ statistic, the corresponding diagonal elements of the plot have non-blue colors. Warm colors on the off-diagonal of the plot indicate that a detachment of the component meta-analysis with design *d* (shown in the columns) reduces the inconsistency at design *d*’ (shown in the rows). The inconsistency between direct and indirect evidence at design *d*’ before the detachment is indicated by the color of the diagonal element *d*’. An increase in inconsistency is indicated by blue colors. The stronger the intensity of the color is, the greater the difference between the inconsistency before and after the detachment of studies with design *d* is. The color of the whole plot is implemented to have a maximum intensity for absolute values greater or equal to eight.

**Figure 2:**
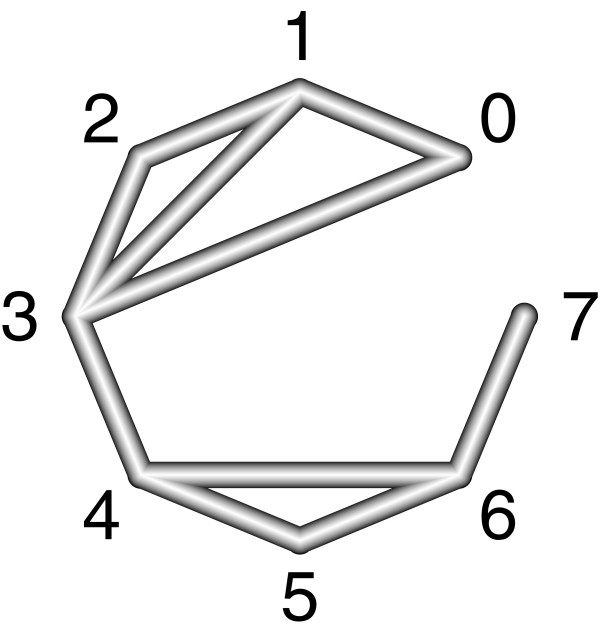
**Network design of an illustrative network meta-analysis.** The nodes correspond to eight treatments and the edges display observed treatment comparisons. Design 6:7 and 3:4 do not contribute to the inconsistency assessment and are not incorporated into a net heat plot.

Designs where only one treatment is involved in other designs of the network (for example design 6:7 in Figure 2) or where the removal of $S_{d}$ would lead to a splitting of the network (for example design 3:4 in Figure 2) do not contribute to the inconsistency assessment and are not incorporated into the net heat plot.

For the arrangement of the rows and columns of the plotted matrix, we use the sum of the absolute distances between the rows and the absolute distances between the columns of ${\left(Q_{d^{\prime },d}^{\text{diff}}\right)}_{d^{\prime },d=1,\ldots ,D}$ for complete linkage clustering (see for example [34]). This results in colored block structures that potentially indicate hot spots of inconsistency.

In the plot we also draw gray squares, as shown in Figure 1b), with areas proportional to the corresponding absolute elements of the hat matrix from equation (6). The larger the square is, the stronger the direct estimate of design *d* drives the network estimate of design *d*’. Consequently, a design *d* with large squared Pearson residuals $Q_{d}^{\text{inc}}$ strongly influences design *d*’. The combination of the color for the inconsistency and the differently-sized squares results in the visual appearance of a halo that relays both types of information at the same time (see for example [35] for use of such halo visualizations in a different context).

### Further illustrative examples

**Figure 3:**
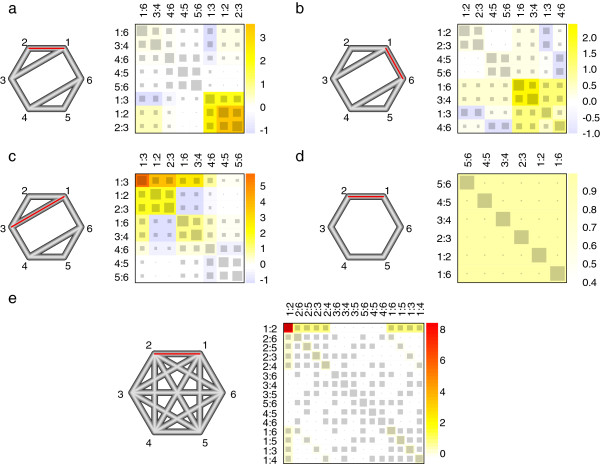
**Five illustrative network meta-analyses with net heat plot.** In **a**) to **e**), the network design is shown on the left: six treatments and six, eight or fifteen different observed designs based on two-armed studies. The nodes are placed on the circumcircle and are labeled according to the treatments. The edges show which treatments are directly compared. The thickness of an edge represents the inverse standard error ${\left(V_{d}^{\text{d}\text{ir}}\right)}^{-1/2}$, which is equal one for all designs. We introduced inconsistency by perturbing the effect of one edge (marked in red) by five standard errors of the direct effect estimate. The corresponding net heat plots are shown on the right side: The area of the gray squares displays the contribution of the direct estimate in design *d* (shown in the column) to the network estimate in design *d*’ (shown in the row). The colors are associated with the change in inconsistency between direct and indirect evidence in design *d*’ (shown in the row) after detaching the effect of design *d* (shown in the column). Blue colors indicate an increase and warm colors indicate a decrease (the stronger the intensity of the color, the stronger the change).

To illustrate the application of the net heat plot, we consider the network example from the previous sections and Figure 1 as well as four additional network meta-analysis examples with six treatments and six, eight, or all possible fifteen component meta-analyses based on two-armed studies (*d* in {1:2, 1:3,…,5:6} with *N*_*d*_=2). These networks are displayed as graphs in Figures 3a) to e) on the left side, where the edges correspond to the different direct comparisons. The thickness of an edge represents the inverse standard error $V_{d}^{-1/2}$, which is equal to one for all d∈;{1,…,D}. We have produced an inconsistent network of treatment effects by adding a *δ*=5 to one treatment effect $\theta_{d}^{\text{dir}}$, while all other effects of the network remain zero.

Because the network structures and the assumed precisions of the direct effects are the same in scenarios a) to c), they share the same hat matrix, which is discussed in Section “Identifying drivers via the hat matrix” and illustrated in Figure 1b). That is why the net heat plots in Figures 3a) to c) contain the same gray squares, just ordered differently due to the clustering.

In scenario a), inconsistency is introduced through the treatment effect in design 1:2. The overall inconsistency statistic is *Q*^inc^=9.17 (*p*=0.027, see Table 2). In the net heat plot, the color intensities of the diagonal elements indicate that the squared Pearson residual for design 1:3 and especially the residuals for the designs 1:2 and 2:3 almost solely contribute to *Q*^inc^. The latter ones have higher residuals, although their direct estimates drive their network estimates more strongly, with 63% in contrast to 53% in the case of design 1:3. This can be seen in the hat matrix elements that are displayed here by the area of the squares. The warm-colored off-diagonal elements in the column of design 1:2 or 2:3 are equal to the colors on the diagonal, which indicates a complete elimination of inconsistency in the whole network after relaxing design 1:2 or 2:3. This is also recognizable by $Q_{\left(1:2\right)}^{\text{inc}}$ and $Q_{\left(2:3\right)}^{\text{inc}}$ in Table 2, each with a p value of one. A detachment of design 1:3 does not reduce all residuals but increases that of the designs 1:6, 3:4, and 4:6, as indicated by the blue colors. Relaxing other designs causes only little change to the squared Pearson residuals. For example relaxing design 1:6 weakly reduces the residuals of design 1:2 and 2:3 but inflates the residuals of design 1:3 and increases the inconsistency in the whole network (*p*=0.016 for $Q_{\left(1:6\right)}^{\text{inc}}$). Due to the arrangement of the rows and columns in the plot (as explained in Section “The net heat plot”), we can see a hot spot of inconsistency between the effects of the component meta-analyses with designs 1:2, 2:3, and 1:3 by the warm-colored block on the diagonal; however, the effect of 1:3 is supported by other evidence of the network shown by the blue-colored elements in row and column of design 1:3. Altogether, designs 1:2 and 2:3 can be identified as a source of inconsistency in the network. However, to be able to understand whether the effects of the component meta-analyses of both designs are the source or whether only one of them is, we need more network connectivity so that they are included solely in network loops. The squares in the columns of the two identified designs show that the corresponding treatment effects drive the network estimate of design 1:3, which is therefore perturbed. Although attenuated, driving is also observed in designs 1:6, 3:4, and 4:6, as far as the influence of the effect in design 1:2 (and 2:3) is sufficient.

**Table 2:** The inconsistency in the illustrative examples

	**a)**			**b)**			**c)**			**d)**			**e)**		
	***Q***	***df***	***p***	***Q***	***df***	***p***	***Q***	***df***	***p***	***Q***	***df***	***p***	***Q***	***df***	***p***
*Q*^inc^	9.17	3	0.027	7.50	3	0.058	11.67	3	0.009	4.17	1	0.041	16.67	10	0.082
$Q_{\left(1:2\right)}^{\text{inc}}$	0.00	2	1	6.82	2	0.033	6.82	2	0.033	0.00	0	1	0.00	9	1
$Q_{\left(1:3\right)}^{\text{inc}}$	5.36	2	0.069	5.36	2	0.069	0.00	2	1				15.62	9	0.075
$Q_{\left(1:4\right)}^{\text{inc}}$													15.62	9	0.075
$Q_{\left(1:5\right)}^{\text{inc}}$													15.62	9	0.075
$Q_{\left(1:6\right)}^{\text{inc}}$	8.33	2	0.016	0.00	2	1	8.33	2	0.016	0.00	0	1	15.62	9	0.075
$Q_{\left(2:3\right)}^{\text{inc}}$	0.00	2	1	6.82	2	0.033	6.82	2	0.033	0.00	0	1	15.62	9	0.075
$Q_{\left(2:4\right)}^{\text{inc}}$													15.62	9	0.075
$Q_{\left(2:5\right)}^{\text{inc}}$													15.62	9	0.075
$Q_{\left(2:6\right)}^{\text{inc}}$													15.62	9	0.075
$Q_{\left(3:4\right)}^{\text{inc}}$	8.33	2	0.016	0.00	2	1	8.33	2	0.016	0.00	0	1	16.67	9	0.054
$Q_{\left(3:5\right)}^{\text{inc}}$													16.67	9	0.054
$Q_{\left(3:6\right)}^{\text{inc}}$													16.67	9	0.054
$Q_{\left(4:5\right)}^{\text{inc}}$	9.09	2	0.011	6.82	2	0.033	11.36	2	0.003	0.00	0	1	16.67	9	0.054
$Q_{\left(4:6\right)}^{\text{inc}}$	8.93	2	0.012	5.36	2	0.069	10.71	2	0.005				16.67	9	0.054
$Q_{\left(5:6\right)}^{\text{inc}}$	9.09	2	0.011	6.82	2	0.033	11.36	2	0.003	0.00	0	1	16.67	9	0.054

The Q statistic for inconsistency as well the Q statistic after detaching the effect of design *d* are given for each of the scenarios a) to e) in Figure 3. In addition, the degrees of freedom (df) of the corresponding chi-squared distribution and the p value (p) are shown.

In scenario b), we shifted the effect in design 1:6 analogously to scenario a) by *δ*=5 from the rest of the network. This causes a *Q*^inc^ of only 7.50 with a *p*=0.058, which is mainly composed of the squared Pearson residuals of designs 1:3 and 4:6 and especially of the residuals of designs 1:6 and 3:4. Contrasting the colors and the size of squares on the matrix diagonal shows that the latter two hold the strongest inconsistency contribution, although their corresponding direct estimates drive their network estimates the most strongly. In this scenario, a detachment of the effect in designs 1:6 or 3:4 eliminates the inconsistency of the network. In contrast, relaxing one of the designs 1:2, 2:3, 4:5, or 5:6 only slightly reduces the inconsistency of the whole network (each $Q_{\left(1:2\right)}^{\text{inc}}$, $Q_{\left(2:3\right)}^{\text{inc}}$, $Q_{\left(4:5\right)}^{\text{inc}}$, $Q_{\left(5:6\right)}^{\text{inc}}$ with *p*=0.033), and a detachment of designs 1:3 or 4:6 even increases the inconsistency (each $Q_{\left(1:3\right)}^{\text{inc}}$, $Q_{\left(4:6\right)}^{\text{inc}}$ with a *p*=0.033). As well, in all six cases the squared Pearson residual of at least one other design is inflated. So in this scenario, we see a hot spot of inconsistency between designs 1:6, 3:4, 1:3, and 4:6 by the intense warm-colored block on the diagonal (4×4). The strongest inconsistency is between the effect in designs 1:6 and 3:4. Weaker inconsistency can be observed between the effects in the designs 1:2 and 2:3 as well between the effects in 4:5 and 5:6. The effects of designs 1:3 and 4:6 are supported by the evidence of the designs 1:2 and 2:3 as well as 4:5 and 5:6 respectively. So in this scenario, designs 1:6 and 3:4 can be identified as a plausible source of inconsistency, and analogous to scenario a), the inconsistency causing edge 1:6 cannot be distinguished from the jointly-acting edges 3:4, although in this example these two are not adjacent edges. The squared Pearson residuals for the two identified designs, shown on the diagonal of the plot, are smaller in comparison to the residuals of the designs 1:2 and 2:3 in scenario a), although in both scenarios a perturbation is introduced with *δ*=5. This is because the corresponding network estimates are more strongly driven by their direct estimates with each 70% and not only with 63% as in designs 1:2 and 2:3. The squares in the columns of the two identified designs indicate that they drive the network estimates in designs 1:3 and 4:6 and, a little weaker, of the remaining other designs, which therefore differ from their direct estimates. Contrasting the colors and the size of squares on the off-diagonal elements of all 2×2 blocks on the diagonal implies that the weakest amount of treatment effect deviation is observed between the effects in designs 1:2 and 2:3 as well as between the effects in 4:5 and 5:6, since the largest hat matrix elements are here as well the less intensive color. Altogether, the influence of the perturbed treatment effect in design 1:6 is more broad, but with overall weaker severity as the equally perturbed effect in scenario a).

In scenario c), we changed the effect in design 1:3 with *δ*=5 and found the highest network inconsistency statistic *Q*^inc^=11.67 (*p*=0.009) in comparison to both previous scenarios. The squared Pearson residual for design 1:3 provides the largest contribution to the *Q*^inc^ statistic. Smaller residuals are observed for the adjacent edges 1:2, 2:3, 1:6, and 3:4. A detachment of the effect in design 1:3 eliminates the inconsistency of the network. Relaxing other designs causes only a little change to the squared Pearson residuals and increases residuals for some designs. A hot spot of inconsistency can be seen between the effects in designs 1:3, 1:2, and 2:3. However, the effect in design 1:2 is supported by the effects in designs 1:6, 3:4, and 4:6, and vice versa, the latter ones are supported by the effects in design 1:2. The same holds for the effect in design 2:3 and the effects in the three designs. Altogether, edge 1:3 can be distinctly identified as a plausible source of inconsistency since this is nested in two loops. The squared Pearson residual for this design is higher in comparison to the residuals for the inconsistency-generating designs in the previous two scenarios, although in all scenarios an equally strong perturbation is introduced. This is because 1:3 is the least self-driving design. Since the effect of design 1:3 strongly drives the network estimates of the designs 1:2, 2:3, 1:6, and 3:4, they are also influenced by the perturbation.

In scenario d), we analyze a sparsely connected network that forms one loop. In such a network with observed inverse standard errors being the same for each direct estimate, all corresponding network estimates are composed 83% of its own and 17% balanced of all other direct estimates. So, in the net heat plot we see only large squares on the diagonal. A perturbation of the effect at design 1:2 results in a network inconsistency statistic of *Q*^inc^=4.17 (*p*=0.041), which is the sum of equally-sized squared Pearson residuals. A detachment of any design interrupts the loop and flow of evidence so that the network estimates correspond, if existing, to their direct estimates and the inconsistency of the network is dissolved. In this scenario, we can recognize inconsistency but cannot locate its source since we have insufficient degrees of freedom. Nevertheless, several indirect estimates were affected by the perturbation of design 1:2.

In network scenario e), all fifteen possible pairwise comparisons are observed with same precision. Because of this tight linkage, each network estimate is driven one-third by its corresponding direct estimate. The remaining two-thirds of indirect estimation is based on all eight adjacent edges in a balanced way. The disturbance of the network consistency by adding a *δ*=5 to treatment effect $\theta_{1:2}^{\text{dir}}$ does not produce as much inconsistency in the whole network as seen in the other scenarios (*Q*^inc^=16.67 with *p*=0.082). Almost exclusively, the squared Pearson residual for design 1:2 is increased so that a detachment of design 1:2 eliminates the inconsistency. A detachment of one of the eight adjacent edges causes only a little change and even weakly increases the inconsistency in the whole network, which results each time in a p value of 0.075. In the case of non-adjacent edges, the p values corresponding to $Q_{\left(d\right)}^{\text{inc}}$ are even 0.054. So in this scenario, the source of inconsistency is uniquely identifiable in the net heat plot, even more easily compared to scenario c). It only weakly drives and affects the network estimates of its adjacent edges so that the perturbation of the effect in design 1:2 has only a little influence on the network.

The examples show that perturbation of a single design may have side effects on residuals, more or less spread out in the network. Our clustering proved successful in grouping together designs with interrelated residuals that were simultaneously affected by one perturbation. The resulting hot spots facilitate the identification of sources of inconsistency, which may or may not be uniquely identifiable. While related large residuals are obviously grouped together, it may also occur that large residuals emerging from two independent perturbations are also grouped in proximity. In this case we expect to find two diagonal blocks, each signaling the local side effects of one perturbation and each representing one hot spot of inconsistency.

### Software

We implemented our methods in the open-source statistical environment R[36]. While multivariate meta-analysis for the aggregation step of studies with the same design can be carried out using standard statistic software [28,37], we provide a preliminary stand-alone R function for the net heat plot available on the website http://www.unimedizin-mainz.de/fileadmin/kliniken/imbei/Dokumente/Biometrie/Software/netheat.R. An R package is in preparation and will be available from the standard CRAN repository for the R environment.

## Results

### An example of a network meta-analysis in diabetes

We applied our methods to a network meta-analysis example by Senn at al. [17]. They examined the continuous outcome of blood glucose change according to the marker HbA1c in patients with type two diabetes after adding one treatment out of ten different groups of glucose-lowering agents to a baseline sulfonylurea therapy. As effect measures, we consider mean differences.

**Figure 4:**
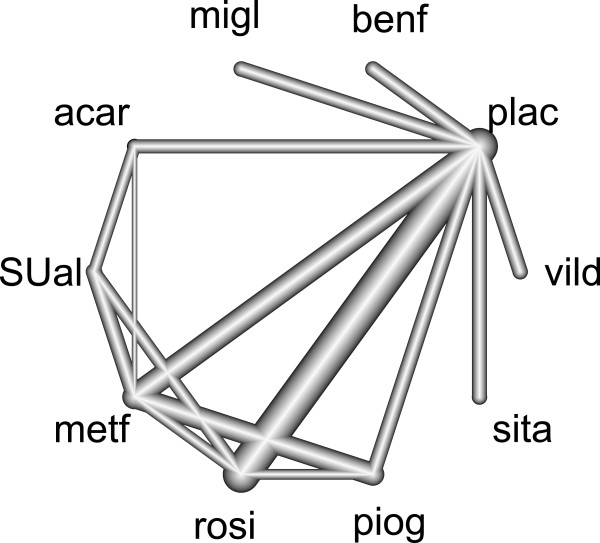
**Network design in the diabetes example.** The nodes are placed on the circumcircle and are labeled according to the treatments. The edges display the observed treatment comparisons. The thickness of the edges is proportional to the inverse standard error of the treatment effects, aggregated over all studies including the two respective treatments. The network includes 25 two-armed studies on fourteen different designs and one three-armed study of design plac:acar:metf.

The ten different treatment groups are abbreviated as follows by their first four letters: acar: Acarbose, benf: Benfluorex, metf: Metformin, migl: Miglitol, plac: Placebo, piog: Pioglitazone, rosi: Rosiglitazone, sita: Sitagliptin, SUal: Sulfonylurea alone, vild: Vildagliptin. This network meta-analysis involved 26 randomized controlled trials including one three-armed trial for plac:acar:metf and 15 different designs, of which ten are used in only one study. In the network, 15 out of 45 possible different pair-wise contrasts are observed, of which eight involve a placebo (see Figure 4).

Across the entire network (analogues to the result of Senn at al. [17]) as well as for exclusively within designs, we observed heterogeneity with p values <0.001 (see Table 3). Regarding the $Q_{d}^{\text{het}}$ statistics, the component meta-analyses with designs plac:benf, plac:metf, plac:migl, and, plac:rosi contribute the most to the heterogeneity within designs.

**Table 3:** Heterogeneity and inconsistency in the diabetes example

**Q statistic**		**Number of studies**	**Degrees of freedom**	**p value**
*Q*^net^	96.98	26	27-9=18	<0.001
*Q*^inc^	22.53	26	16-9=7	0.002
*Q*^het^	74.45	26	27-16=11	<0.001
$Q_{\text{plac:benf}}^{\text{het}}$	4.38	2	2-1=1	0.036
$Q_{\text{plac:metf}}^{\text{het}}$	42.16	3	3-1=2	<0.001
$Q_{\text{plac:migl}}^{\text{het}}$	6.45	3	3-1=2	0.040
$Q_{\text{plac:rosi}}^{\text{het}}$	21.27	6	6-1=5	0.001
$Q_{\text{metf:rosi}}^{\text{het}}$	0.19	2	2-1=1	0.665

The decomposition of the Q statistics as well as the degrees of freedom of the corresponding chi-squared distributions and the p values are shown. In addition the considered number of studies are displayed. Only one study is observed for the following designs: plac:acar, plac:piog, plac:sita, plac:vild, acar:SUal, metf:piog, metf:SUal, piog:rosi, rosi:SUal, plac:acar:metf. For this reason, the corresponding $Q_{d}^{\text{het}}$ statistics are not shown.

**Figure 5:**
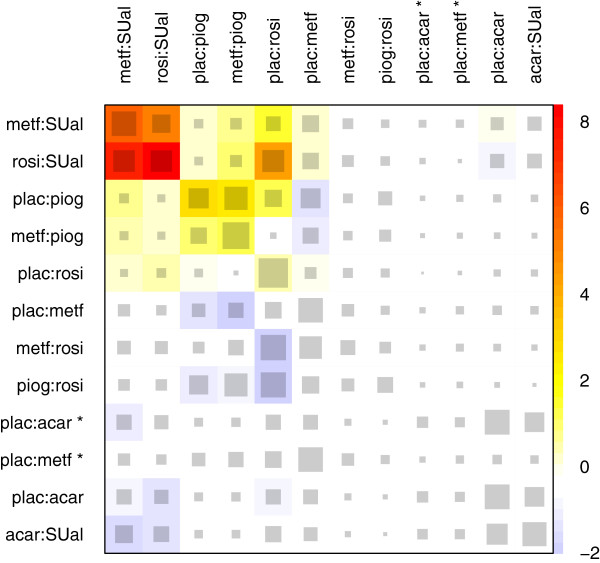
**Net heat plot in the diabetes example.** The area of the gray squares displays the contribution of the direct estimate in design *d* (shown in the column) to the network estimate in design *d*’ (shown in the row). The colors are associated with the change in inconsistency between direct and indirect evidence in design *d*’ (shown in the row) after detaching the effect of design *d* (shown in the column). Blue colors indicate an increase and warm colors indicate a decrease (the stronger the intensity of the color, the stronger the change). The two contrasts of the three-armed study with design plac:acar:metf are marked with ^∗^.

To have a closer look at the inconsistency of the network, we use the net heat plot in Figure 5. Studies with design plac:benf, plac:migl, plac:sita, or plac:vild are not included in this plot because they do not contribute to the inconsistency assessment. There are direct treatment effects that strongly drive other network estimates in a consistent manner. For example, the treatment effects in designs plac:acar and acar:SUal agree with the existing direct evidence of each other, but we observe a *Q*^inc^ statistic with a p value of 0.002, which is composed of the squared Pearson residuals for the designs metf:SUal, rosi:SUal, plac:piog, metf:piog, and plac:rosi. The first two have higher residuals in comparison to plac:piog, although their direct estimates more strongly drive their network estimates, with 56% and 41% in contrast to 36% in the case of design plac:piog. We can observe a hot spot of inconsistency between the effects in designs metf:SUal, rosi:SUal, plac:piog, and metf:piog, for which only one study is observed in each case. The effects in designs plac:piog and metf:piog as well as, in particular, the designs metf:SUal and rosi:SUal are especially inconsistent. Although the direct estimate in design plac:rosi is hampered with large heterogeneity (*p*=0.001), it has a large evidence base of six studies and hence strongly drives its network estimate with 83% and other network estimates as well. Note, that the contribution of single studies is easily disclosed by splitting the amount of 83% into a sum according to the inverse variances of the estimates of each study (83*%*=11*%*+18*%*+20*%*+22*%*+4*%*+8*%*). A detachment of the corresponding design reduces the residuals of design metf:SUal, rosi:SUal, and plac:piog, but inflates the residuals of design metf:rosi and piog:rosi. Overall, a detachment of the effects for each of the five inconsistent component meta-analyses mentioned increases the squared Pearson residuals for some other designs in the network and results in blue entries in the plot.

**Figure 6:**
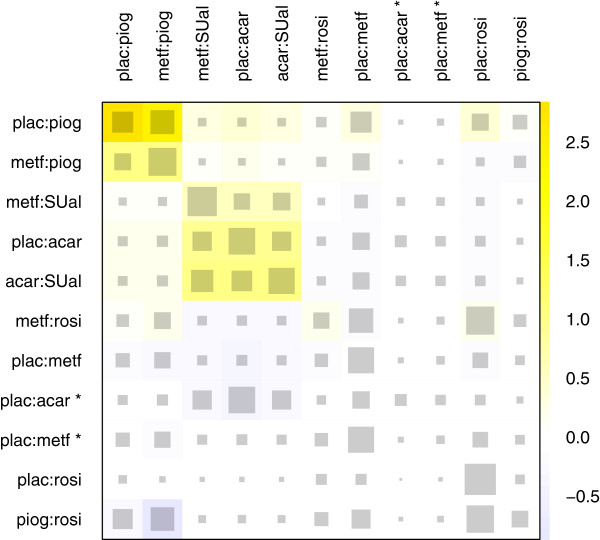
**Net heat plot in the diabetes example after exclusion of the study with design rosi:SUal.** The area of the gray squares displays the contribution of the direct estimate in design *d* (shown in the column) to the network estimate in design *d*’ (shown in the row). The colors are associated with the change in inconsistency between direct and indirect evidence in design *d*’ (shown in the row) after detaching the effect of design *d* (shown in the column). Blue colors indicate an increase and warm colors indicate a decrease (the stronger the intensity of the color, the stronger the change). The two contrasts of the three-armed study with design plac:acar:metf are marked with ^∗^.

The strongest reduction in the whole network inconsistency is achieved with a detachment of the effect in design rosi:SUal. In this case, the net heat plot in Figure 6 results. The inconsistency between the effects in designs plac:piog and metf:piog remains, but in an attenuated form. Now, the effect of design metf:SUal is inconsistent with the effect of the designs plac:acar and acar:SUal, which were supported by the effect in design rosi:SUal in the previous version of the network. However, with a p value of 0.342 for the *Q*^inc^ statistic, there is no longer strong evidence for inconsistency. The hot spot of inconsistency detected included designs with only one study. Indeed, one or a few biased studies may either cause heterogeneity when paralleled by other studies of the same design (which is observed within the plac:rosi studies) or may cause inconsistency when solely representing a design.

### An example of a network meta-analysis in antidepressants

**Figure 7:**
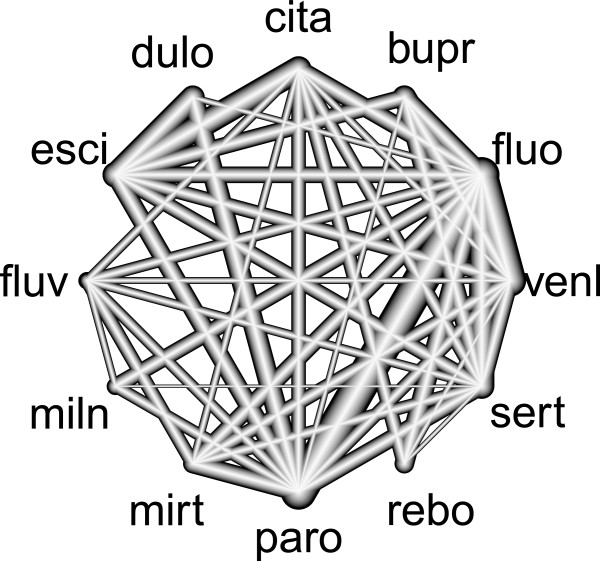
**Network design in the antidepressants example.** The nodes are placed on the circumcircle and are labeled according to the treatments. The edges display the observed treatment comparisons. The thickness of the lines is proportional to the inverse standard error of the treatment effect, aggregated over all studies including these two respective treatments. The network includes 109 two-armed studies with 42 different designs and two three-armed studies, both with design fluo:paro:sert.

Cipriani et al. [12] performed a network meta-analysis to examine the efficacy between twelve new-generation antidepressants as monotherapy for the acute-phase treatment of major depression. The twelve antidepressants are abbreviated as follows: bupr: Bupropion, cita: Citalopram, dulo: Duloxetine, esci: Escitalopram, fluo: Fluoxetine, fluv: Fluvoxamine, miln: Milnacipran, mirt: Mirtazapine, paro: Paroxetine, rebo: Reboxetine, sert: Sertraline, venl: Venlafaxine. The efficacy was defined as a reduction of at least 50% from the baseline depression rating score after 8 weeks. For the network meta-analysis, they involved 111 randomized controlled trials including two three-armed trials of design fluo:paro:sert. In these studies, 42 of 66 possible pair-wise contrasts between the 12 treatments are observed (see Figure 7) in *D*=43 different designs, of which 16 are observed in only one study.

Analogous to [12], we used log odds ratios as effect measures, but for combining study estimates we used the fixed-effects model (5) instead of a random-effects model within the Bayesian framework. The treatment effects and respective standard errors of our model are very similar to the results of Cipriani et al. [12], and the standard errors are not systematically smaller as could be expected, because we observed only little heterogeneity in the whole network (*p*=0.113) as well as within designs (*p*=0.125) and no significant inconsistency (*p*=0.293). This results from the calculated *Q* statistics corresponding to Section “Decomposition of Cochran’s Q” (see Table 4). Regarding the heterogeneity within the designs, only the two studies with design paro:sert are conspicuous, with a p value of 0.006.

**Table 4:** Heterogeneity and inconsistency in the antidepressants example

**Q statistic**		**Number of studies**	**Degrees of freedom**	**p value**
*Q*^net^	119.6	111	113-11=102	0.113
*Q*^inc^	36.9	111	44-11=33	0.293
*Q*^het^	82.7	111	113-44=69	0.125

The Q statistic for the whole network, for inconsistency and for heterogeneity within designs are shown. In addition, the number of studies on which they are based, the degrees of freedom of the corresponding chi-squared distributions and the corresponding p values are displayed.

**Figure 8:**
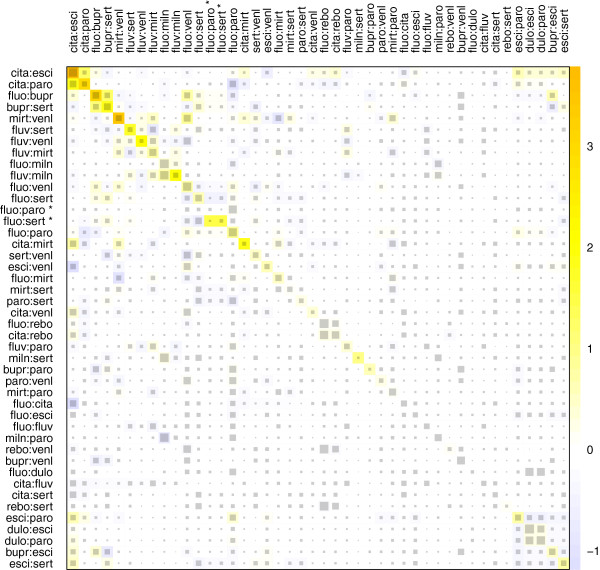
**Net heat plot in the antidepressants example.** The area of the gray squares displays the contribution of the direct estimate in design *d* (shown in the column) to the network estimate in design *d*’ (shown in the row). The colors are associated with the change in inconsistency between direct and indirect evidence in design *d*’ (shown in the row) after detaching the effect of design *d* (shown in the column). Blue colors indicate an increase and warm colors indicate a decrease (the stronger the intensity of the color, the stronger the change). The two contrasts of the two three-armed trials with design fluo:paro:sert are marked with ^∗^.

The net heat plot presented in Figure 8 provides a detailed assessment of the slight inconsistency in this quite tightly connected network. As seen from the color on the diagonal of the plot, the squared Pearson residuals for designs cita:esci, cita:paro, fluo:bupr, and mirt:venl contribute the most to *Q*^inc^. There is a small hot spot of inconsistency between the effects in designs cita:esci and cita:paro as well as between the effects in fluo:bupr and bupr:sert. The largest squared Pearson residual is observed for design cita:esci, although the direct estimate in this design drives the corresponding network estimate comparatively strongly with 51% (maximum self-driving is observed in design dulo:esci with 61%). In contrast to the other four designs mentioned, the direct estimate of cita:esci also strongly drives network estimates for some other designs in the network, which can be seen from the square sizes in the corresponding column. A detachment of the effect in design cita:esci results in the strongest reduction of the inconsistency in the whole network (resulting in $Q_{\left(\text{cita}:\text{esci}\right)}^{\text{inc}}=29.6$ with *p*=0.591). While the direct evidence contributes more than 50% of the network estimate of this contrast, the direct estimate is larger than the network estimate (log odds ratio 0.39 vs. 0.17), and publication bias may be affecting the former one. The squared Pearson residuals for the designs cita:paro, cita:mirt, esci:paro, and esci:sert are particularly reduced. In contrast, the direct treatment effects of designs fluo:venl and fluo:paro have the smallest standard error and drive the network estimates of many other designs (see large squares in the corresponding columns in Figure 8); however, a detachment of one of these designs causes only small changes in the squared Pearson residuals.

## Discussion

To ensure the validity and robustness of the conclusion from a network meta-analysis, it is important to assess the consistency of the network and the contribution of each component meta-analysis to the estimates. Our intention was to develop a sensitivity analysis tool that allows the identification of which component meta-analyses drive which network estimates and to locate the drivers that may have generated a hot spot of inconsistency. The net heat plot serves both purposes simultaneously: the first one by graphically showing elements of the hat matrix and the latter one by colored block structures in the plot. We have shown that the net heat plot allows the identification of a single deviating design that induces inconsistency in artificial examples. In the case of stronger network connectivity, increased location specificity might be possible. In networks that only include one loop, it is not possible to trace inconsistency back to a single design, but designs that are part of several loops may be identifiable as a unique source for a hot spot of inconsistency. We also demonstrated the applicability of the plot in two published network meta-analyses.

It is well known in regression diagnostics (see for example [29]) that the influence of an observation (on parameter estimates and prediction) is driven by both the respective residual and the diagonal element of the hat matrix. Analogous to classical meta-analyses, outlier effect estimates of single studies or a few highly-weighted studies play an important role, which can be inspected in forest plots. Influence measures are usually displayed as index plots with observation numbers on the horizontal axis; this has been successfully exploited for simple meta-analysis [21,22]. We felt that this is insufficient in network meta-analyses and thus proposed the net heat plot as an additional tool. We display all elements of the hat matrix in the net heat plot and pointed out that the lines of the hat matrix are the linear coefficients for a specific network estimate. As such they represent the natural generalization of simple meta-analysis weights. They quantify the contribution of a component meta-analysis to the network-estimate of a given contrast and may therefore be of interest, even in a consistent network meta-analysis. Simultaneously, the changes in the squared Pearson residuals are visualized in the net heat plot after allowing for a deviating effect of one single component meta-analyses to detect outlying direct estimates. In passing, we have shown that Cochran’s chi-squared statistic, the sum of squared Pearson residuals, can be generally used in network meta-analyses in a fixed-effects model framework to assess the heterogeneity of the whole network and can be decomposed to separate out the inconsistency of the network. Particularly, we have shown how multi-armed studies can be incorporated both into the inconsistency chi-squared statistic via a quadratic form of Pearson residuals and into the net heat plot.

Overall, inconsistency testing has also been discussed in large complex networks by comparing a consistency model with an unrestricted inconsistency model [10], as we have done in turn for each single component meta-analysis. However, the authors essentially only consider inconsistency between two-armed component meta-analyses because they do not analyze independent effects for multi-armed studies. We included multi-armed studies and thereby opened a way for dividing overall heterogeneity exhaustively into heterogeneity within designs and inconsistency. Within a Bayesian framework, the authors discuss models with and without a random component for heterogeneity within component meta-analyses. We advocate the fixed-effects model, not only for the sake of simplicity but, more importantly in the diagnostic framework, because it potentially provides a clearer picture and allows for better recognition and location of inconsistency. In contrast to testing loops for inconsistency [6], which leads to redundant testing of many dependent hypotheses or is confined to simple networks composed of independent loops (as argued in [10]), our approach is applicable in large and complex networks. The approaches that capture inconsistency by a single extra variance component in a mixed effects model [8,9] only aim at quantifying inconsistency and at providing conservative confidence intervals. The assumptions are difficult to justify or falsify and, more importantly, the approach contains no straightforward way to locate inconsistency.

The recently published design-by-treatment interaction model by [15,16] is most similar in spirit to our approach. In contrast to White et al. [16] and Higgins et al. [15], we do not include random effects for heterogeneity within designs. The advantage is that one or a few deviating or biased studies are treated equally, whether they are paralleled by many other studies of the same design or are the sole representative of their design. In a random-effects model, the former studies would add to the heterogeneity variance whereas the latter studies would inform fixed design-by-treatment interaction parameters. In a fixed-effects model, inconsistency is indicated by the *Q* statistic more sensitively than in the random-effects model of [16]. If heterogeneity or inconsistency is detected and not explained by single outliers, the model should be extended with study level covariates, along the lines explored by [6,38]. Ideally we should end up with a homogeneous model, thereby explaining rather than modeling heterogeneity and inconsistency.

However, the fact that failure to detect heterogeneity does not constitute proof of homogeneity must be taken into account in the assessment of inconsistencies in network meta-analyses. This already holds for a simple meta-analysis and is even more relevant for network meta-analyses. In a network without loops, inconsistency cannot be detected at all. In this context, we point to the importance of the hat matrix. It allows for the assessment of the contribution of each component meta-analysis to a network estimate and directs attention to the crucial components. We have illustrated that often only a few components are important.

Often, when inconsistency is observed, some component meta-analyses are heterogeneous, too. We point out that the inconsistency assessment is still valid in this context. However, then the direct effect estimates are no longer estimates of a single parameter, but are rather weighted averages of estimates of different parameters: the study-specific treatment effects. Nevertheless, inconsistency assessment and the investigation of heterogeneity within component meta-analyses may interfere in this case, and it may be necessary to exclude single studies and repeat the net heat plot in order to find satisfactory explanations of overall heterogeneity. In fact, inspection of both coefficients (entries of the hat matrix) and of residuals was proposed by Senn et. al [17] at the study level, and this may be more appropriate if heterogeneity within designs is large. However, when applied at the study level, the net heat plot also has the additional advantage of pointing to influential studies, i.e. studies with large weight and large residuals.

Heterogeneity and inconsistency can be broadly viewed as different aspects of heterogeneity, the latter being understood as any discrepancy between results of single studies and predictions based on a consistency model for a network. This fact is not only reflected in the decomposition of the Q statistic, but also underlines that our tools can be applied either at an aggregate level or at a study level. We presented the aggregate level approach here for its parsimony. The study level approach may be more appropriate, particularly if component meta-analyses are strongly heterogeneous. In fact, a visual display of the hat matrix at study level has been proposed and discussed in [17]. Another potentially viable approach would be to complement our tools at an aggregate level with ordinary forest plots for component meta-analyses.

Some caution is due when interpreting a net heat plot. Different from usual regression diagnostics, a single component meta-analysis may stand for a large body of evidence in network meta-analyses. If a component meta-analysis is recognized as deviating from the rest or is identified as a major source of heterogeneity, it may or may not provide the more reliable part of the whole body of evidence. Song et al. [39] argued that sometimes the indirect part of evidence may be more reliable than the direct part. That is why tracking heterogeneity should only be the starting point for focusing on subject matter details of component meta-analyses and, hopefully, single studies for finding subject matter reasons for the observed heterogeneity, as argued by [40] for classical meta-analyses. In fact, this process of investigation was demonstrated in one example without using a formal tool to sort out inconsistency; this was a simple inspection of squared Pearson residuals [17] and has been elaborated upon in worked examples (e.g. in [38,41]). In large and complex networks, we feel that the two step approach, separately investigating inconsistency and heterogeneity within designs is necessary in order to limit efforts. Furthermore it specifically can answer whether a set of studies sharing the same design is influential.

More than in classical regression diagnostics, there are model diagnostic challenges in network meta-analyses: Masking, a phenomenon already known, may be more pronounced here because we have inherently small numbers of observations: the component meta-analyses. Masking may occur if more than one observation deviates from the true model. In this case, parameter estimates are affected by outliers even after holding out one observation, and outliers may be obscured, i.e. masked [29]. To tackle this, we combined the technique of withholding one observation with a graphical display. While this is clearly adequate if only one outlier exists, it may also facilitate the detection of more outliers. For a more rigorous approach, methods of holding out several observations will have to be explored. The second problem, uniqueness, is particularly virulent in network meta-analyses: several component meta-analyses could be the explanation for all observed inconsistency. We discussed the extreme case of a circular network where inconsistency is completely unidentifiable. The ability to track down inconsistency to only one or at least a few component meta-analyses depends, as we illustrated, on the connectedness of the network. A lack of network connectivity can be useful for planning further studies, but the challenges for future research are twofold: find rules for the identifiability of deviating components and to find tools for economically displaying the ambiguity if it exists.

Searching for influential component meta-analyses or influential studies is not the only way for responding to inconsistency and heterogeneity. As mentioned in [16] and worked out in [38], the consistency model can be extended to allow for (treatment by covariate interaction of) study level covariates, and the model extension can explain inconsistency and heterogeneity. Both approaches are complementary. Of note, the net heat plot could again be applied to an extended consistency model.

One core component of our approach is to allow component meta-analyses to have deviating treatment effects. This idea of extending the model by relaxing parameter constraints is easily extended to generalized linear models for binary outcomes as well as to random-effects models. The approach is not confined to withholding the effects of one design, but is naturally applicable to allowing for an arbitrary number of designs to have specific deviating effects, e.g. all designs containing a specific treatment. In all types of generalization, the challenge remains to perform these model relaxations in a systematic way and to provide tools to transparently display the multitude of results, for which our presented net heat can be a useful starting point.

## Conclusions

We have illustrated the importance of assessing consistency in network meta-analyses, where, for example, one deviating component meta-analysis may induce a hot spot of inconsistency. As a tool for this task, we have developed the net heat plot that displays drivers of the network estimates, plausible sources for inconsistency, and possible disturbed network estimates, illustrating its usefulness in several artificial and real data examples.

## Competing interests

The authors declare that they have no competing interests.

## Authors’ contributions

UK, HB and JK developed the method. UK produced the results and wrote the first draft of the manuscript. HB and JK contributed to the writing. All authors read and approved the final manuscript.

## Pre-publication history

The pre-publication history for this paper can be accessed here:

http://www.biomedcentral.com/1471-2288/13/35/prepub

## References

[B1] Wells GA Sultan SA Chen L Khan M Coyle D (Eds) Indirect Evidence: Indirect Treatment Comparisons in Meta-Analysis 2009 Ottawa: Canadian Agency for Drugs and Technologies in Health

[B2] Hoaglin DC Hawkins N Jansen JP Scott DA Itzler R Cappelleri JC Boersma C Thompson D Larholt KM Diaz M Barrett A Conducting indirect-treatment-comparison and network-meta-analysis studies: report of the ISPOR task force on indirect treatment comparisons good research practices: part 2 Value Health 2011 14 4 429 437 [http://dx.doi.org/10.1016/j.jval.2011.01.011] 10.1016/j.jval.2011.01.011 21669367

[B3] Dias S Welton NJ Sutton AJ E AA (Eds) A Generalised Linear Modelling Framework for Pairwise and Network Meta-Analysis of Randomised Controlled Trials, 2011 NICE DSU: Technical Support Document 2 [http://www.nicedsu.org.uk] 27466657

[B4] Salanti G Indirect and mixed-treatment comparison, network, or multiple-treatments meta-analysis: many names, many benefits, many concerns for the next generation evidence synthesis tool Res Syn Meth 2012 3 2 80 97 [http://doi.wiley.com/10.1002/jrsm.1037] 10.1002/jrsm.1037 26062083

[B5] Baker SG Kramer BS The transitive fallacy for randomized trials: if A bests B and B bests C in separate trials, is A better than C? BMC Med Res Methodol 2002 2 13 10.1186/1471-2288-2-13 12429069 PMC137603

[B6] Salanti G Marinho V Higgins JPT A case study of multiple-treatments meta-analysis demonstrates that covariates should be considered J Clin Epidemiol 2009 62 8 857—864 [http://dx.doi.org/10.1016/j.jclinepi.2008.10.001] 19157778 10.1016/j.jclinepi.2008.10.001

[B7] Jorgensen AW Maric KL Tendal B Faurschou A Gotzsche PC Industry-supported meta-analyses compared with meta-analyses with non-profit or no support: differences in methodological quality and conclusions BMC Med Res Methodol 2008 8 60 [http://dx.doi.org/10.1186/1471-2288-8-60] 10.1186/1471-2288-8-60 18782430 PMC2553412

[B8] Lumley T Network meta-analysis for indirect treatment comparisons Stat Med 2002 21 16 2313—2324 [http://dx.doi.org/10.1002/sim.1201] 12210616 10.1002/sim.1201

[B9] Lu G Ades AE Assessing evidence inconsistency in mixed treatment comparisons J Am Stat Assoc 2006 101 474 447 459 10.1198/016214505000001302

[B10] Dias S Welton NJ Sutton AJ Caldwell DM Guobing L Ades AE (Eds) Inconsistency in Networks of Evidence Based on Randomised Controlled Trials, 2011 NICE DSU: Technical Support Document 4 [http://www.nicedsu.org.uk] 27466656

[B11] Bucher HC Guyatt GH Griffith LE Walter SD The results of direct and indirect treatment comparisons in meta-analysis of randomized controlled trials J Clin Epidemiol 1997 50 6 683 691 10.1016/S0895-4356(97)00049-8 9250266

[B12] Cipriani A Furukawa TA Salanti G Geddes JR Higgins JP Churchill R Watanabe N Nakagawa A Omori IM McGuire H Tansella M Barbui C Comparative efficacy and acceptability of 12 new-generation antidepressants: a multiple-treatments meta-analysis Lancet 2009 373 9665 746 758 [http://dx.doi.org/10.1016/S0140-6736(09)60046-5] 10.1016/S0140-6736(09)60046-5 19185342

[B13] Salanti G Higgins JPT Ades AE Ioannidis JPA Evaluation of networks of randomized trials Stat Methods Med Res 2008 17 3 279 301 [http://dx.doi.org/10.1177/0962280207080643] 17925316 10.1177/0962280207080643

[B14] Dias S Welton NJ Caldwell DM Ades AE Checking consistency in mixed treatment comparison meta-analysis Stat Med 2010 29 7–8 932 944 [http://dx.doi.org/10.1002/sim.3767] 20213715 10.1002/sim.3767

[B15] Higgins JPT Jackson D Barrett JK Lu G Ades aE White IR Consistency and inconsistency in network meta-analysis: concepts and models for multi-arm studies Res Syn Meth 2012 3 2 98 110 [http://doi.wiley.com/10.1002/jrsm.1044] 10.1002/jrsm.1044 PMC4433772 26062084

[B16] White IR Barrett JK Jackson D Higgins JPT Consistency and inconsistency in network meta-analysis: model estimation using multivariate meta-regression Res Syn Meth 2012 3 2 111 125 [http://doi.wiley.com/10.1002/jrsm.1045] 10.1002/jrsm.1045 PMC4433771 26062085

[B17] Senn S Gavini F Magrez D Scheen A Issues in performing a network meta-analysis Stat Methods Med Res 2012 (Epub ahead of print). [http://dx.doi.org/10.1177/0962280211432220] 10.1177/0962280211432220 22218368

[B18] Rücker G Network meta-analysis, electrical networks and graph theory Res Syn Meth 2012 3 4 312 324 [http://doi.wiley.com/10.1002/jrsm.1058] 10.1002/jrsm.1058 26053424

[B19] Caldwell DM Welton NJ Ades AE Mixed treatment comparison analysis provides internally coherent treatment effect estimates based on overviews of reviews and can reveal inconsistency J Clin Epidemiol 2010 63 8 875 882 [http://dx.doi.org/10.1016/j.jclinepi.2009.08.025] 10.1016/j.jclinepi.2009.08.025 20080027

[B20] Chatterjee S Hadi AS Influential Observations, High Leverage Points, and Outliers in Linear Regression Statist Sci 1986 1 3 379 393 10.1214/ss/1177013622

[B21] Viechtbauer W Cheung WL Outlier and influence diagnostics for meta-analysis Res Syn Meth 2010 1 2 112 125 10.1002/jrsm.11 26061377

[B22] Gumedze FN Jackson D A random effects variance shift model for detecting and accommodating outliers in meta-analysis BMC Med Res Methodol 2011 11 19 [http://dx.doi.org/10.1186/1471-2288-11-19] 10.1186/1471-2288-11-19 21324180 PMC3050872

[B23] Lu G Welton NJ Higgins JPT White IR Ades A Linear inference for mixed treatment comparison meta-analysis: A two-stage approach Res Syn Meth 2011 2 43 60 10.1002/jrsm.34 26061599

[B24] Chung H Lumley T Graphical exploration of network meta-analysis data: the use of multidimensional scaling Clin Trials 2008 5 4 301 307 [http://dx.doi.org/10.1177/1740774508093614] 10.1177/1740774508093614 18697844

[B25] Galbraith RF A note on graphical presentation of estimated odds ratios from several clinical trials Stat Med 1988 7 889 894 10.1002/sim.4780070807 3413368

[B26] Aitken AC On least squares and linear combination of observations Proc R Soc Edinb 1934 55 42 48

[B27] Gleser LJ Olkin I Cooper H, Hedges LV, Valentine JC Stochastically dependent effect sizes The Handbook of Research Synthesis and Meta-Analysis, 2009 New York: Russell Sage Foundation 357 376

[B28] Jackson D Riley R White IR Multivariate meta-analysis: Potential and promise Stat Med 2011 30 20 2481 2498 [http://www.ncbi.nlm.nih.gov/pmc/articles/PMC3470931/] 10.1002/sim.4172 PMC3470931 21268052

[B29] Belsley DA Kuh E Welsch RE Regression Diagnostics: Identifying Influential Data and Sources of Collinearity (Wiley Series in Probability and Statistics) 2004 New Jersey: John Wiley & Sons Ⓒ1980

[B30] Cochran W The combination of estimates from different experiments Biometrics 1954 10 101 129 10.2307/3001666

[B31] Raudenbush SW Becker BJ Kalaian H Modeling multivariate effect sizes Psych Bull 1988 103 111 120

[B32] Borenstein M Hedges LV Higgins JPT Rothstein HR Introduction to Meta-Analysis 2009 Chichester: John Wiley & Sons

[B33] Song F Clark A Bachmann MO Maas J Simulation evaluation of statistical properties of methods for indirect and mixed treatment comparisons BMC Med Res Meth 2012 12 138 [http://www.ncbi.nlm.nih.gov/pubmed/22970794] 10.1186/1471-2288-12-138 PMC3524036 22970794

[B34] Gordon AD Classification 1999 London: Chapman and Hall/ CRC

[B35] Oelke D Janetzko H Simon S Neuhaus K Keim D Visual boosting in pixel-based visualizations Comput Graphics Forum 30 2011 3 871—880

[B36] R Core Team R: A Language and Environment for Statistical Computing 2012 Vienna: R Foundation for Statistical Computing [http://www.R-project.org/]. [ISBN 3-900051-07-0]

[B37] Gasparrini A Armstrong B Kenward MG Multivariate meta-analysis for non-linear and other multi-parameter associations Stat Med 2012 31 29 3821 3839 10.1002/sim.5471 22807043 PMC3546395

[B38] Cooper NJ Sutton AJ Morris D Ades AE Welton NJ Addressing between-study heterogeneity and inconsistency in mixed treatment comparisons: Application to stroke prevention treatments in individuals with non-rheumatic atrial fibrillation Stat Med 2009 28 14 1861 1881 10.1002/sim.3594 19399825

[B39] Song F Harvey I Lilford R Adjusted indirect comparison may be less biased than direct comparison for evaluating new pharmaceutical interventions J Clin Epidemiol 2008 61 5 455 463 [http://www.ncbi.nlm.nih.gov/pubmed/18394538] 10.1016/j.jclinepi.2007.06.006 18394538

[B40] Thompson SG Why sources of heterogeneity in meta-analysis should be investigated BMJ 1994 309 6965 1351 1355 10.1136/bmj.309.6965.1351 7866085 PMC2541868

[B41] Salanti G Dias S Welton NJ Ades AE Golfinopoulos V Kyrgiou M Mauri D Ioannidis JPA Evaluating novel agent effects in multiple-treatments meta-regression Stat Med 2010 29 23 2369 2383 [http://dx.doi.org/10.1002/sim.4001] 20687172 10.1002/sim.4001

